# Balloon Test Occlusion of Internal Carotid Artery in Recurrent Nasopharyngeal Carcinoma Before Endoscopic Nasopharyngectomy: A Single Center Experience

**DOI:** 10.3389/fonc.2021.674889

**Published:** 2021-07-06

**Authors:** Renhao Yang, Hui Wu, Binghong Chen, Wenhua Sun, Xiang Hu, Tianwei Wang, Yubin Guo, Yongming Qiu, Jiong Dai

**Affiliations:** Department of Neurosurgery, Renji Hospital, School of Medicine, Shanghai Jiao Tong University, Shanghai, China

**Keywords:** balloon test occlusion, recurrent nasopharyngeal carcinoma, endoscopic nasopharyngectomy, near-infrared spectroscopy cerebral oximeter, regional cerebral oxygen saturation, cerebral collateral circulation

## Abstract

**Objectives:**

Endoscopic nasopharyngectomy (ENPG) is a promising way in treating recurrent nasopharyngeal carcinoma (rNPC), but sometimes may require therapeutic internal carotid artery (ICA) occlusion beforehand. Balloon test occlusion (BTO) is performed to evaluate cerebral ischemic tolerance for ICA sacrifice. However, absence of neurological deficits during BTO does not preclude occur of delayed cerebral ischemia after permanent ICA occlusion. In this study, we evaluate the utility of near-infrared spectroscopy (NIRS) regional cerebral oxygen saturation (rSO_2_) monitoring during ICA BTO to quantify cerebral ischemic tolerance and to identify the valid cut-off values for safe carotid artery occlusion. This study also aims to find out angiographic findings of cerebral collateral circulation to predict ICA BTO results simultaneously.

**Material and Methods:**

87 BTO of ICA were performed from November 2018 to November 2020 at authors’ institution. 79 angiographies of collateral flow were performed in time during BTO and classified into several Subgroups and Types according to their anatomic and collateral flow configurations. 62 of 87 cases accepted monitoring of cerebral rSO_2_. Categorical variables were compared by using Fisher exact tests and Mann–Whitney U tests. Receiver operating characteristic curve analysis was used to determine the most suitable cut-off value.

**Results:**

The most suitable cut-off △rSO_2_ value for detecting BTO-positive group obtained through ROC curve analysis was 5% (sensitivity: 100%, specificity: 86%). NIRS rSO_2_ monitoring wasn’t able to detect BTO false‐negative results (*p* = 0.310). The anterior Circle was functionally much more important than the posterior Circle among the primary collateral pathways. The presence of secondary collateral pathways was considered as a sign of deteriorated cerebral hemodynamic condition during ICA BTO. In Types 5 and 6, reverse blood flow to the ICA during BTO protected patients from delayed cerebral ischemia after therapeutic ICA occlusion (*p* = 0.0357). In Subgroup IV, absence of the posterior Circle was significantly associated with BTO-positive results (*p* = 0.0426).

**Conclusion:**

Angiography of cerebral collateral circulation during ICA BTO is significantly correlated with ICA BTO results. Angiographic ICA BTO can be performed in conjunction with NIRS cerebral oximeter for its advantage of being noninvasive, real-time, cost-effective, simple for operation and most importantly for its correct prediction of most rSO_2_ outcomes of ICA sacrifice. However, in order to ensure a safe carotid artery occlusion, more quantitative adjunctive blood flow measurements are recommended when angiography of cerebral collateral circulation doesn’t fully support rSO_2_ outcome among clinically ICA BTO-negative cases.

## Introduction

Nasopharyngeal carcinoma (NPC) features itself by its distinct geographical distribution, particularly prevails in east and southeast Asia (1). Radiotherapy, combined with or without chemotherapy, is the primary treatment modality for initially untreated NPC (2). However, it is also noteworthy that about 10–20% of NPC patients have suffered from local recurrence on follow-up after primary treatment (3). The management of recurrent nasopharyngeal carcinoma (rNPC) is challenging, while re-irradiation and endoscopic nasopharyngectomy (ENPG) serve as the two mainstays of the treatment of rNPC (4, 5). Radiotherapy of recurrent locoregional tumor mass has reached the bottleneck among rNPC patients owing to its high rate of severe complications such as osteoradionecrosis, temporal lobe necrosis, multiple cranial nerve dysfunction and potentially fatal bleeding, which can greatly impair patients’ quality of life and occasionally result in death (6). In comparison to re-irradiation, endoscopic surgical resection is a new, promising and better way in treating selected rNPC patients in terms of locoregional control rate and overall survival (OS) rate with lower incidence of long-term severe complications (2).

However, preoperative safety management of internal carotid artery (ICA) is of vital importance before surgery, because ICA bleeding can be catastrophic during the operation, especially for tumors invading the ICA. In fact, patients harboring head and neck tumors may require therapeutic occlusion of ICA before tumor resection as preoperative preparation (7). Permanent occlusion of ICA is a useful procedure, but carries the risk of severe and irreversible complications caused by immediate or delayed hemodynamic cerebral ischemia (8). Balloon test occlusion (BTO) of ICA is performed to evaluate cerebral ischemic tolerance for the purpose of reducing neurological ischemic complications after permanent occlusion of ICA among these patients (9). Awareness of the reliance of ICA among patients is quite essential, because covered stent implantation or vascular bypass might be needed for patients when ICA sacrifice is not tolerated (10–12). Scholars have demonstrated that ischemic complication rate for unselected ICA occlusion without BTO reached 26%, with 12% mortality rate related to the cerebral infraction, and was reduced to 13% when BTO was performed (13). However, several previous studies have also revealed that BTO alone still carried a false-negative risk of 3.3–10.0% (13, 14). That is why adjunctive techniques are used in combination with BTO to increase its sensitivity (15). However, most of the blood flow measurements proposed in addition to the basic method require specialized equipment, thus increasing the complexity and perioperative complications due to the extended inflation time of the balloon (16).

Meanwhile, blood flow obstructed during BTO of ICA can be recruited from other places, depending on the development of collateral pathways including the Circle of Willis (CoW) (17). Hence, we speculate that some of the ICA BTO results can be predicted by angiographic findings of cerebral collateral circulation.

The present study reviewed our institutional experience in a simple paradigm combining clinical tolerance with monitoring of regional cerebral oxygen saturation (rSO_2_) and angiographic crossflow assessment. In this study, we evaluated the utility of near-infrared spectroscopy (NIRS) cerebral oximeter, which could calculate and monitor rSO_2_ in real time during ICA BTO, to quantify ischemic tolerance and to identify the valid cut-off values for safe carotid artery occlusion. This study also tried to find out some angiographic findings of cerebral collateral circulation to predict ICA BTO results, which might help to choose a more suitable and individualized adjunctive measurement during BTO (18).

## Material and Methods

### Patients

From November 2018 to November 2020, 81 consecutive patients (59 males and 22 females, mean age: 52.5 years old, range from 28 to 70 years old) who underwent BTO for ICA on the lesion side were enrolled in the study. Apart from 81 patients, another six patients didn’t manage to perform ICA BTO, because they were identified with chronic internal carotid artery occlusion (CICAO) on the lesion side after DSA confirmation before BTO (**Supplemental Table 4**). All patients had a clear diagnosis of rNPC and those with distant metastasis were excluded.

### Balloon Test Occlusion Procedure

Informed consent was obtained from every patient. The probes of NIRS cerebral oximeter (MC-2030C cerebral oximeter, CAS MEDICAL SYSTEMS Inc., USA) were placed on the forehead of both sides, and rSO_2_ was monitored continuously during all procedures. The whole procedure was performed under local anesthesia. The patients were wide awake and were aware that the operator would continuously communicate with them to evaluate his or her motor function, sensory system, speech, orientation and cognition during the procedure.

All procedures were performed with single plane DSA equipment, Innova 3100-IQ (GE Inc., USA) and Artis Q Zeego (Simens Inc., Germany). After local anesthesia induction, a 6-French femoral sheath was inserted into the right femoral artery. After that, control anteroposterior and lateral angiography of bilateral ICAs and unilateral vertebral artery (VA) were performed at six frames per second before BTO with a diagnostic catheter. Heparin was administered intravenously after a puncture to prevent procedure-related thromboembolic complications. A nondetachable balloon catheter was then introduced and placed in the distal cervical segment of ICA on the lesion side through the balloon guiding catheter. The balloon positioned in the lower segment of the ICA might lead to bradycardia and a transient drop of blood pressure (BP) caused by carotid sinus reflex, which might affect the result of BTO.

After performing the initial neurological evaluation, the balloon (Sterling Monorail, Boston Scientific Inc., USA) was then inflated under fluoroscopic visualization with angiographic confirmation of complete flow arrest of the vessel. The tested ICA was occluded for a total duration of 30 min and a neurological assessment was repeated immediately after the occlusion and every 5 min during the test. If the patient didn’t show with sign of the symptoms caused by hemodynamic cerebral ischemia 15 min after the temporary occlusion of ICA, the operator would puncture the left femoral artery and performed angiography of the contralateral ICA and the unilateral VA to evaluate the collateral circulation with the balloon inflated. As soon as any neurological deficit with rSO_2_ abnormality was detected during the procedure, the balloon was deflated immediately regardless of the collateral angiography. Under such circumstance, the BTO was considered positive. Once the patient clinically tolerated the 30-min occlusion, the BTO was judged to be negative. Patient developing delayed cerebral ischemia after therapeutic ICA occlusion was judged to be false-negative.

### Post-Occlusion Protocol

Hemostasis at the femoral punctures were treated by electronic compressor. After permanent ICA occlusion by coiling, patients were transferred to the intensive care unit (ICU) where fluid balance, neurologic status, and blood pressure were carefully monitored. Subcutaneous heparin at therapeutic dosages was continued for 48 h. Routine head CT scanning was performed 24 h after carotid occlusion. Attention was focused on detecting symptoms of cerebral ischemia that might have been caused by the occlusion of a carotid artery. BP monitoring was another concern after the operation. BP should be kept pharmacologically elevated for 48 h to avoid possible episode of hypotension if necessary. Early stage of permanent blockage of the blood flow to the ICA might lead to an increased pressure within the carotid sinus, hence resulting in a temporary drop in BP and subsequent craniocerebral hypoperfusion. If no delayed cerebral hemodynamic ischemia was detected, the patients were discharged 5 days after the treatment and then transferred to EYE & ENT hospital of Fudan University for salvage ENPG.

### Anatomic Configuration and Collateral Flow Configuration

It is widely acknowledged that the CoW exhibits considerable anatomical variations (19–21). A complete CoW only accounts for approximately 50% of the population (19, 20, 22). The anterior Circle of CoW is composed of the anterior communicating artery (AcomA) and the proximal anterior cerebral artery (ACA) segments (A1) (23). Asymmetry of anterior cerebral artery trunks (A1), with one side A1 (larger diameter) as the dominant supply to both pericallosal arteries (A2) is a common anatomic variation within the anterior part of the CoW and is regarded as an important factor in the formation of aneurysms of the AcomA by producing different hemodynamic stress (24). For another, blood flow through a vessel can be modeled mathematically by using the Pouseille equation relating flow (25). F; to vessel length, L; the pressure drops across the vessel, ΔP; the blood viscosity, η; and the vessel diameter, d:

$$\text{F}\propto \frac{\Delta P\cdot d^{4}}{\eta \cdot L}$$

Under this theory, for two vessels of similar length and pressure, a two times of difference in vessel diameter leads to a sixteen times of difference in blood flow. The Pouseille equation shows that vessel diameter influences the blood supply significantly.

Thus, we speculate that relative position of tested side ICA and dominant A1 will have a great impact on collateral blood flow recruitment and subsequently affect results of BTO. In the presence of AcomA, the A1 to A2 flow dominance was classified on a 3-point scale: symmetric, no clear dominance of the inflow contribution of one A1 segment over the other; dominant, one A1 segment clearly contributes more inflow to an A2 than the other; and complete, no detectable inflow contribution from the contralateral segment. As for the posterior Circle of CoW, it is composed of the posterior communicating arteries (PcomA) and the P1 segments of the posterior cerebral arteries (PCA) (23). Similarly, absence of PcomA and complete fetal PCA (cfPCA), who completely originates from the ICA with no connection with the basilar artery, are other common variants of cerebral circulation (26).

**Figure 1:**
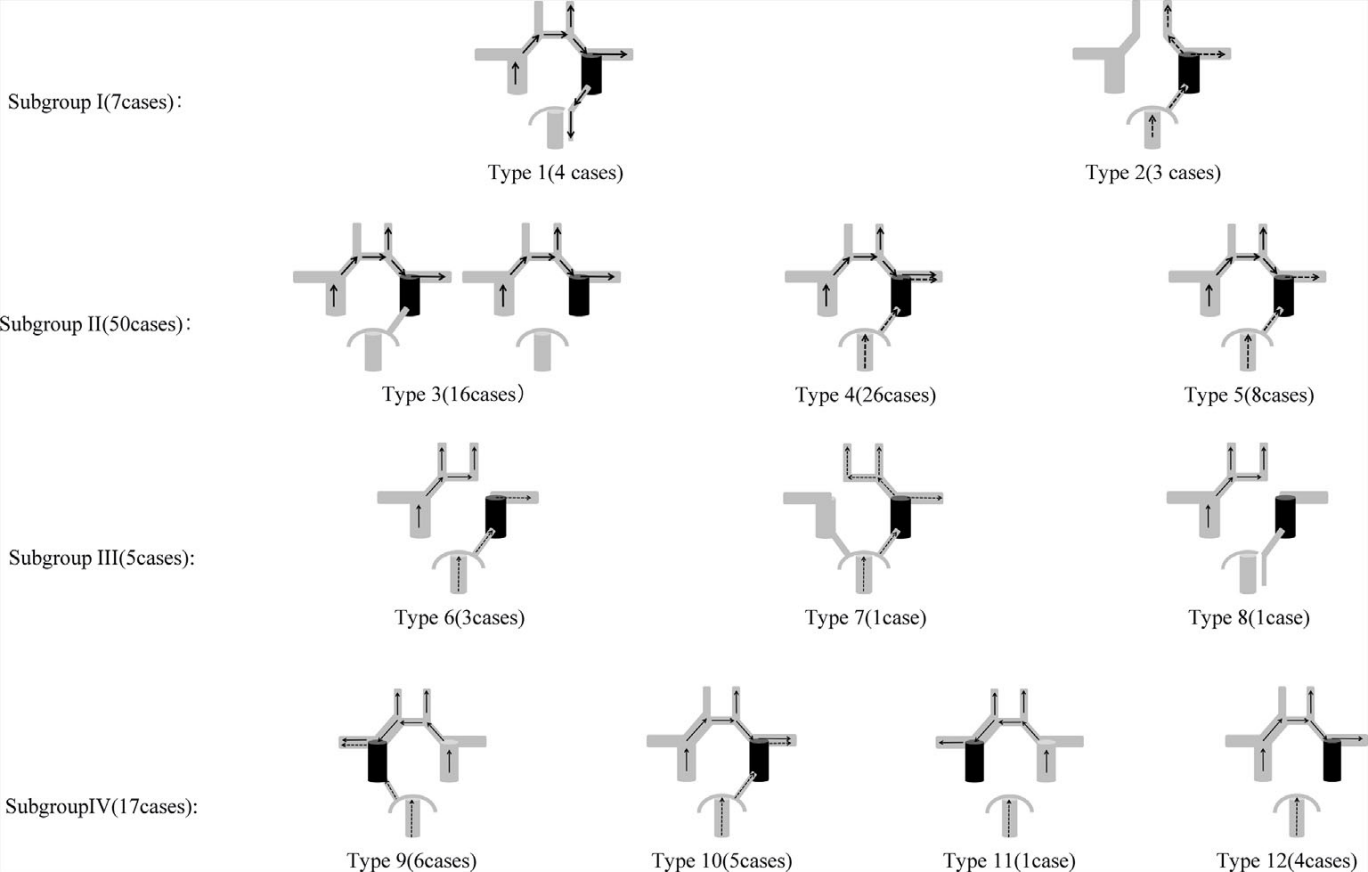
Anatomic Configurations and Collateral Flow Configurations included in this study. Subgroup I: Type 1: A1 to A2 flow dominance: symmetric, cfPCA ipsilateral to tested side; Type 2: AcomA absence, complete posterior Circle ipsilateral to tested side. Subgroup II: A1 to A2 flow dominance: symmetric. Type 3: Tested side MCA area blood supply: the anterior Circle alone; Type 4: Tested side MCA area blood supply: both the anterior Circle and the posterior Circle; Type 5: Tested side MCA area blood supply: the posterior Circle alone. Subgroup III: A1 to A2 flow dominance: complete. Type 6: A1 absence ipsilateral to the tested side; Type 7: A1 absence contralateral to the tested side; Type 8: A1 absence ipsilateral to the tested side, cfPCA ipsilateral to tested side. Subgroup IV: A1 to A2 flow dominance: dominant. Type 9: A1 dominance ipsilateral to the tested side, complete posterior Circle on tested side; Type 10: A1 dominance contralateral to the tested side, complete posterior Circle on tested side; Type 11: A1 dominance ipsilateral to the tested side, PcomA absence on tested side; Type 12: A1 dominance contralateral to the tested side, PcomA absence on tested side.

In our study, the CoW anatomical variations were categorized into several Subgroups (**Figure 1**) as follows based on the presence/absence of AcomA, PcomA and cfPCA, as well as the asymmetry/symmetric of anterior cerebral artery trunks (A1). The Subgroups represented different anatomic configurations of CoW and the Types represented different collateral flow configurations during BTO. The Subgroups and Types only included those we found in this study.

### Statistical Analysis

Continuous variables were reported as mean with standard deviations (SD) or median with interquartile ranges (IQR). Categorical variables were compared by using Fisher exact tests and Mann–Whitney U tests. Receiver operating characteristic (ROC) curve analysis was used to determine the most suitable cut-off value based on the shortest distance from the curve to the upper-left corner. The analysis was performed using SPSS Version 25.0 and Medcalc Version 19.0. The level of significance was established at a 0.05 level (two-sided).

## Results

87 BTO of ICA were performed, as six patients underwent BTO of bilateral ICAs (left: 48 cases; right: 39 cases). 17 cases were judged to be BTO-positive, three cases were judged to be BTO false-negative and the remaining 67 cases were judged to be BTO-negative. Clinical features of all 87 cases were given in **Supplemental Table 1**.

### Cerebral rSO_2_ Monitoring

**Figure 2:**
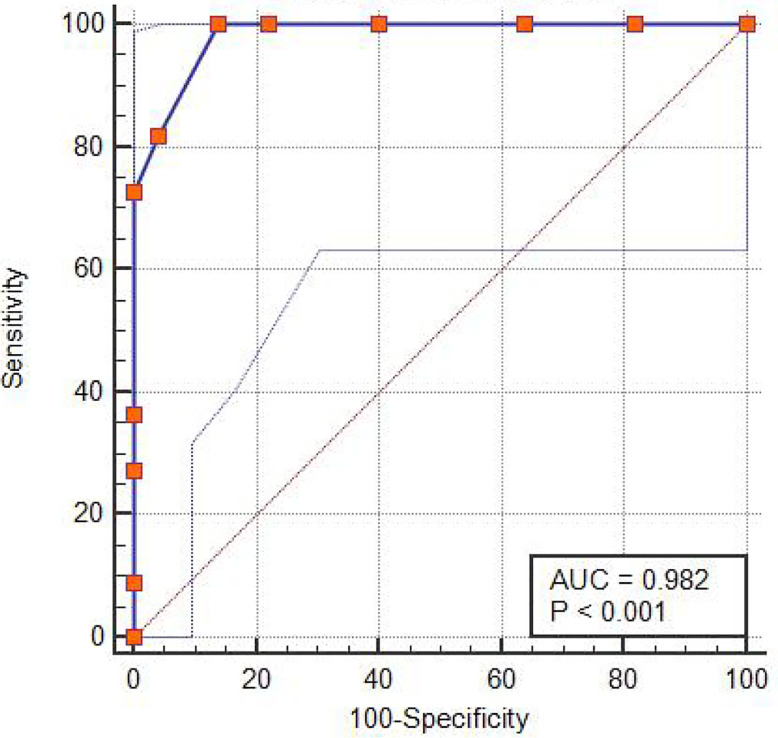
ROC curve analysis for the BTO-positive group. The purple line with orange dots showing ROC curve analysis for the BTO-positive group detected by drop in rSO_2_ value.

62 of 87 cases accepted real-time monitoring of cerebral rSO_2_, among which 12 cases were BTO-positive, 47 cases were BTO-negative and three cases were BTO false-negative. One BTO-positive case (Case No. 77) was excluded in ROC curve analysis because he was found significantly increase in rSO_2_, as the patient complained of unbearable headache during the procedure. Decrease in muscle strength of the right limb (Grade 3/5) was also detected by our clinician. The increase in rSO_2_ was considered to be attributed to increased blood pressure caused by the pain, and the subsequent angiography of the collateral circulation showed insufficient perfusion of the tested side MCA area. The remaining 11 BTO-positive demonstrated significant drop in tested side rSO_2_. As for the decrease in rSO_2_, there was no significant difference between BTO false-negative group (2; 3; 4%) and BTO negative group (median, 2%; IQR, 2%) (*p* = 0.310). In our study, the most suitable cut-off △rSO_2_ value for detecting the BTO-positive group obtained through ROC curve analysis was 5% (sensitivity: 100%, specificity: 86%) (**Figure 2**).

### Angiographic Findings

79 of 87 cases performed complete collateral angiography of contralateral ICA and unilateral VA. Case No. 58 performed complete angiography of collateral circulation, but was excluded from all Types for his unique collateral flow configuration. 79 of 87 cases were included in Subgroups and Types based on their anatomic configurations and collateral flow configurations (**Figure 1**). Case No. 80 failed to perform complete angiography of collateral circulation, but was classified in Subgroup IV based on his anatomic configuration.

#### Subgroup I

A total of seven cases were classified in Subgroup I, among which four cases of tested side cerebral hemisphere blood supply were completely recruited *via* the anterior Circle alone (Type 1) and three cases of tested side cerebral hemisphere blood supply were completely recruited *via* the posterior Circle alone (Type 2). No immediate or delayed hemodynamic cerebral ischemia complications were found in cases in Type 1, while two cases among Type 2 were BTO-positive, but with no statistical difference of ischemic complication rates between two types (*p* = 0.143).

#### Subgroup II

All types had a complete anterior Circle in this subgroup and A1 to A2 blood flow showed no clear dominance of the inflow contribution of one A1 segment over the other. The classification in Subgroup II was based on the different blood supply to the tested side MCA area. Tested side ACA area of all cases in Subgroup II has already gained enough blood perfusion (ASITN/SIR Grade 4) (27) *via* the anterior Circle. Type 3 represented cross flow *via* the anterior Circle alone to the tested side MCA area, whereas Type 4 represented cross flow *via* both anterior Circle and posterior Circle to the tested side MCA area. Type 6 in Subgroup III and Type 5 had similar cerebral hemodynamic features during ICA BTO as they both represented cross flow *via* the posterior Circle alone to the tested side MCA area. The summary of tested side MCA area blood supply categorization and its BTO results for BTO alone group and therapeutic ICA occlusion group are given in **Tables 1** and **2**. All cases in Types 3 and 4 were BTO-negative and no patient suffered delayed ischemia after therapeutic ICA occlusion. When dividing the tested side MCA area blood supply into those with collateral flow *via* the posterior Circle alone (Type 5 +Type 6) and those with collateral flow *via* the anterior Circle alone (Type 3)/via both anterior Circle and posterior Circle (Type 4), a higher BTO-positive rate (33% vs 0%; *p* = 0.046) and BTO false-negative rate (40% vs 0%, *p* = 0.028) in (Type 5 + Type 6) were observed, compared with (Type 3 + Type 4). Cross flow *via* the posterior Circle alone to the tested side MCA area was significantly associated with cerebral ischemic complications (*p* = 0.0108; OR:51; 95% CI: 2.4810 to 1,048.3629).

**Table 1:** The summary of tested side MCA area blood supply categorization in (Subgroup II + Type 6) and its BTO results for BTO alone group.

BTO Alone			
Tested Side MCA Area Blood Supply	BTO-negative (%) (n = 24)	BTO-positive (%) (n = 2)	Total (%) (n = 26)
AC alone	6 (23)	0 (0)	6 (23)
PC alone	4 (15)	2 (8)	6 (23)
AC + PC	14 (54)	0 (0)	14 (54)
Total	24 (92)	2 (8)	26 (100)

AC, the anterior Circle; PC, the posterior Circle.

**Table 2:** The summary of tested side MCA area blood supply categorization in (Subgroup II + Type 6) and its BTO results for Therapeutic ICA Occlusion group.

Therapeutic ICA Occlusion			
Tested Side MCA Area Blood Supply	BTO-negative (%)(n = 25)	BTO false-positive (%)(n = 2)	Total (%)(n = 27)
AC alone	10 (37)	0 (0)	10 (37)
PC alone	3 (11)	2 (7)	5 (19)
AC + PC	12 (44)	0 (0)	12 (44)
Total	25 (93)	2 (7)	27 (100)

AC, the anterior Circle; PC, the posterior Circle.

In cases among Types 5 and 6, we found that no patient experienced cerebral ischemia complications during and after BTO when the sign of reverse blood flow to the ICA was detected (**Table 3**). When we compared the groups with reverse blood flow to the ICA and the groups without reverse blood flow to the ICA during BTO, the odds of having a BTO-negative result given the presence of reverse blood flow to the ICA were 39.0 times greater, with the 95% CI, 1.2772 to 1,190.9128 (*p* = 0.0357).

**Table 3:** The summary of the sigh of reverse blood flow to ICA tested side MCA area blood supply in (Type 5 + Type 6) and its BTO results.

Tested Side MCA Area Blood Supply: PC			
Reverse Blood Flow to ICA (+/−)	BTO-negative (%)(n = 7)	BTO-positive + BTO false-positive (%)(n = 2 + 2)	Total (%)(n = 11)
Presence (+)	6 (55)	0 (0)	6 (55)
Absence (−)	1 (9)	4 (36)	5 (45)
Total	7 (64)	4 (36)	11 (100)

PC, the posterior Circle.

#### Subgroup III

Subgroup III showed no evidence of A1 segment on one side, with total supply to both A2s from the single A1. In Subgroup III, Type 6 was classified into **Tables 1** and **2** and analysis. Only one case (Case No. 62), whose absent A1 was contralateral to the tested side ICA, was categorized in Type 7. This patient underwent BTO alone, with no rSO_2_ abnormality detected during the BTO procedure simultaneously. So the case was considered to be BTO-negative, although angiographic findings showed cross flow not only *via* the posterior Circle to the affected hemisphere but also pial collaterals from contralateral MCA to contralateral ACA.

#### Subgroup IV

In Subgroup IV, all types had a complete anterior Circle and one A1 segment clearly contributed more inflow to an A2 than the contralateral segment. A total of 17 cases were classified in Subgroup IV, among which four cases were BTO-positive, 12 cases were BTO-negative and one case was BTO false-negative. Comparing BTO-negative group and BTO-positive group in Subgroup IV (1 BTO-positive case (Case No. 80) with severe and immediate neurological deficits was excluded, because angiography of collateral blood flow failed to complete in time), the absence of the posterior Circle was significantly associated with BTO-positive results (*p* = 0.0426; OR:29.4000; 95%CI:1.1190–772.4193) (**Table 4**). Statistical analysis showed that relative position of A1 (dominant side) and ICA (tested side) had nothing to do with the configurations of blood supply to tested side MCA area in BTO-negative group in Subgroup IV (*p* = 0.470) (**Supplemental Table 2**). The remaining BTO false-negative case (Case No. 69) was classified in Type 9 based on its hemodynamic change during BTO, but the stenosis of contralateral ICA was detected.

**Table 4:** The summary of the relationship between the absence of Posterior Circle and its BTO results in Subgroup IV.

Posterior Circle (+\−)	BTO-positive (%)(n = 3)	BTO-negative (%)(n = 12)	Total (%)(n = 15)
Presence (+)	0 (0)	10 (67)	10 (67)
Absence (−)	3 (20)	2 (13)	5 (33)
Total	3 (20)	12 (80)	15 (100)

### Case Illustration

**Figure 3:**
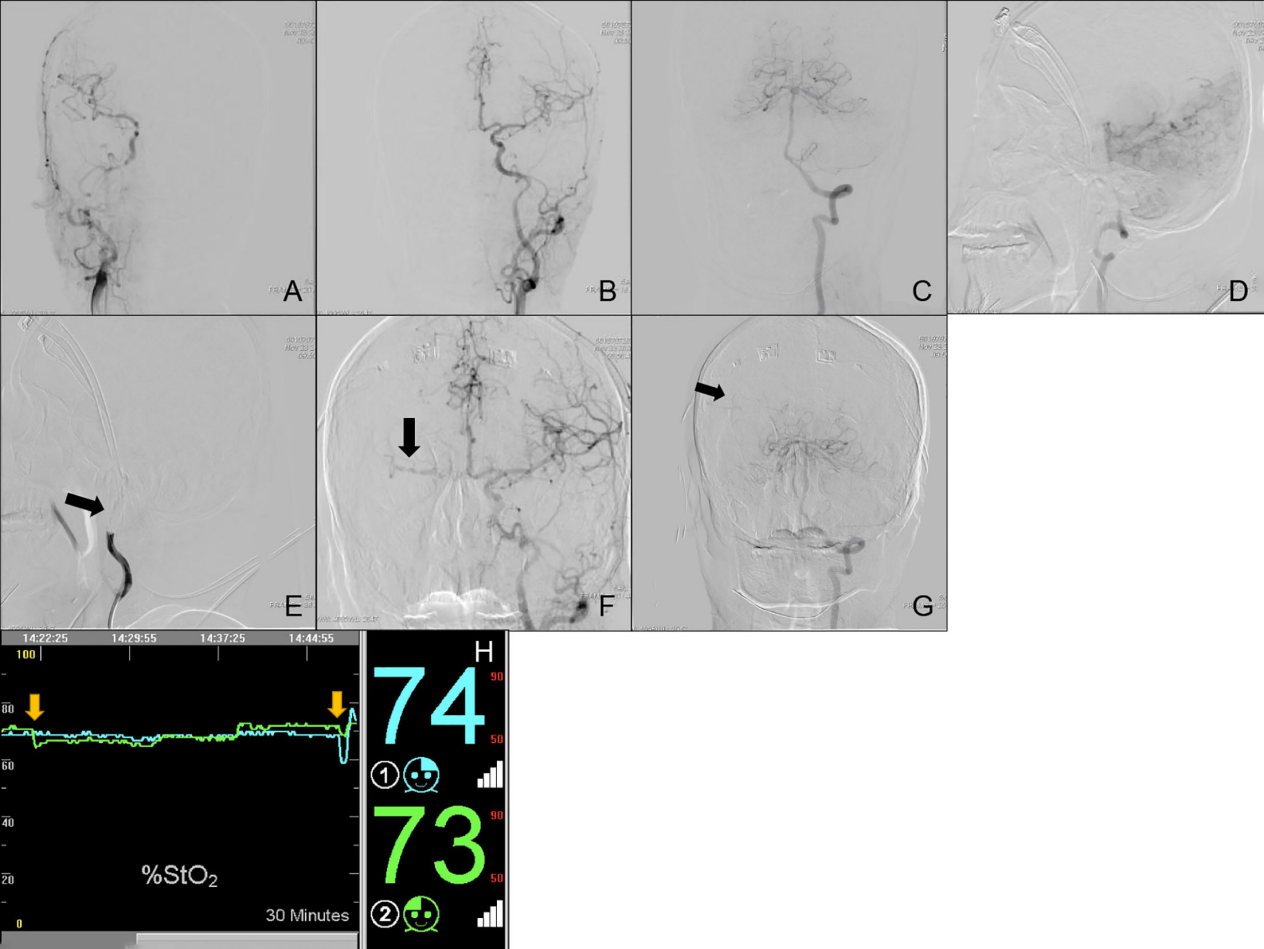
Cerebral angiography and cerebral rSO_2_ monitoring of Case Nos. 76 and 80. **(A–G)** Multi-side view of cerebral angiography. **(H)** Real-time rSO_2_ monitoring. The blue line represented rSO_2_ on the left cerebral hemisphere, the green line represented rSO_2_ on the right cerebral hemisphere, the yellow arrow indicates a sudden drop in rSO_2_.

**Case Nos. 76 and 80**: A 63-year old male (Subgroup IV) was admitted to our neurosurgery department for bilateral ICA BTO and intended to have his left ICA occluded permanently. Control angiography of bilateral ICA and unilateral VA was shown in **Figures 3A–D**. Asymmetry of bilateral A1s was detected in this patient, with left A1 as the dominant side (**Figures 3A, B, F**). **Figure 3E** showed complete temporary blood flow blockage of right ICA (ipsilateral to dominant A1). When BTO of right ICA (contralateral to dominant A1) went on, the patient didn’t show obvious neurological deficits at the beginning. However, rSO_2_ did decrease by 8%, as soon as the balloon was inflated (**Figure 3H**). Subsequent angiography of left ICA showed that insufficient perfusion *via* the anterior Circle to right MCA area during right ICA BTO (ASITN/SIR Grade1) (**Figure 3F**). Angiography of VA showed absence of right PcomA and minor collateral flow *via* leptomengningeal branches from posterior Circulation to the tested side MCA area (**Figure 3G**). The patient gradually became lethargic and demonstrated reduction in muscle strength of the right limb. Then we deflated the balloon in time and the patient recovered immediately. Right ICA BTO was classified into Type 12. When we performed BTO of left ICA, the patient demonstrated loss of consciousness instantly after the balloon was inflated and rSO_2_ dropped more significantly by 11% (**Figure 3H**). Given the severe symptoms caused by cerebral ischemia, we were not able to perform angiography of collateral flow this time. The patient was considered as bilateral BTO-positive and finally underwent covered stent implantation of left ICA.

**Figure 4:**
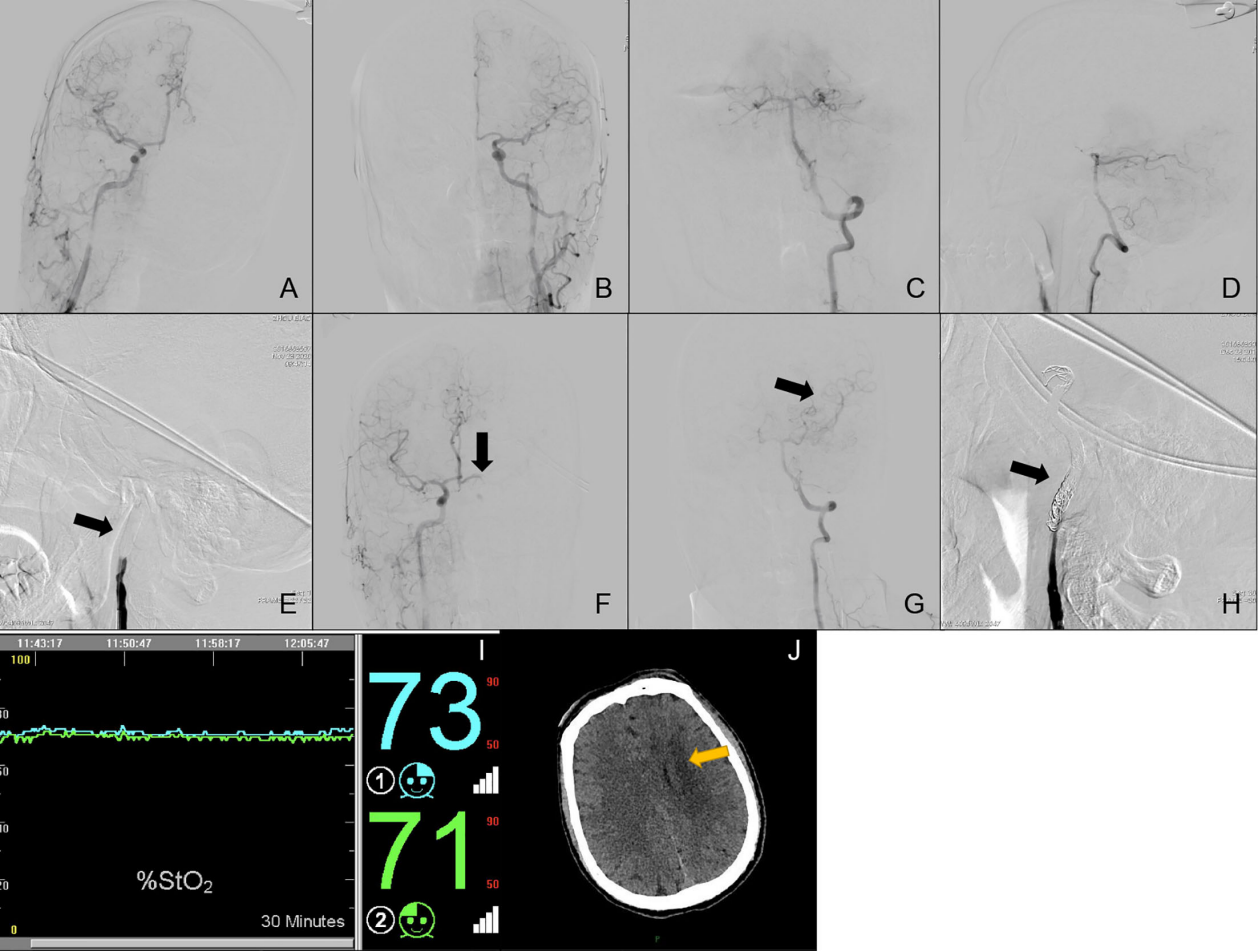
Cerebral angiography and cerebral rSO_2_ monitoring of Case No. 50. **(A–H)** Multi-side view of cerebral angiography. **(I)** Real-time rSO_2_ monitoring. The blue line represented rSO_2_ on the left cerebral hemisphere, the green line represented rSO_2_ on the right cerebral hemisphere. **(J)** CT scan of Case No. 50 after BTO and ICA occlusion, the yellow arrow indicates delayed cerebral infraction after therapeutic ICA occlusion.

**Case No. 50**: A 42-year old male (Subgroup II, Type 5) was admitted to our neurosurgery department for left ICA BTO and intended to have his left ICA occluded permanently. Control angiography of bilateral ICA and unilateral VA was shown in **Figures 4A–D**, with bilateral A1s independently supplying their own A2s (**Figures 4A, B**). **Figure 4E** showed complete temporary blood flow blockage of left ICA. The blood flow from the right ICA only provided enough perfusion for left ACA area (ASITN/SIR Grade4) (**Figure 4F**). Angiography of VA demonstrated that left MCA area (ASITN/SIR Grade3) was supplied by the posterior Circulation *via* the posterior Circle and no reverse blood flow to the left ICA was found (**Figure 4G**). Not any acute cerebral ischemic symptom was detected during BTO. Meanwhile, rSO_2_ monitoring was stable and the value of rSO_2_ fluctuated subtly around the baseline (**Figure 4I**). The patient was considered as BTO-negative at that time and underwent therapeutic ICA occlusion after the test (**Figure 4H**). However, he unfortunately presented loss of muscle strength of right limb and aphasia hours later. Subsequent head CT scanning show cerebral fraction in the left basal ganglia (**Figure 4J**).

**Figure 5:**
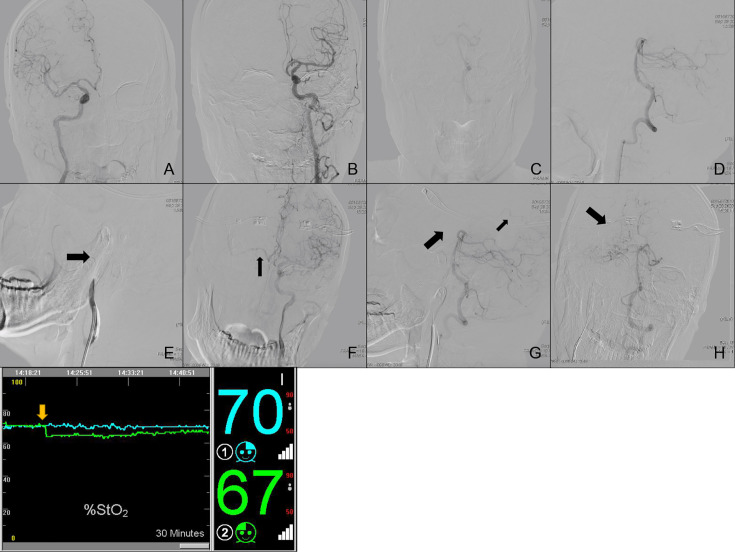
Cerebral angiography and cerebral rSO_2_ monitoring of Case No. 58. **(A–H)** Multi-side view of cerebral angiography. **(I)** Real-time rSO_2_ monitoring. The blue line represented rSO_2_ on the left cerebral hemisphere, the green line represented rSO_2_ on the right cerebral hemisphere, the yellow arrow indicates a sudden drop in rSO_2_.

**Case No. 58:** A 61-year old male was admitted to our neurosurgery department for right ICA BTO. Since he was treated with percutaneous dilational tracheostomy (PDT) not long before and was wearing a tracheostomy tube, he wasn’t able to communicate with us verbally. Control angiography of bilateral ICA and unilateral VA was similar to Case No. 50 (**Figures 5A–D**). **Figure 5E** showed complete temporary blood flow blockage of right ICA. The blood flow from the left ICA only provided enough perfusion for right ACA area (ASITN/SIR Grade4) and showed almost no perfusion to the right MCA area (ASITN/SIR Grade0) (**Figure 5F**). The patient developed moderate collateral flow *via* leptomengningeal branches from posterior Circulation to the MCA area without the presence of right PcomA (**Figures 5G, H**). rSO_2_ on the right side decreased significantly by 7% and fluctuated far below the baseline (**Figure 5I**). The clinician didn’t detect obvious symptoms of cerebral ischemia, but the patient seemed to be unresponsive during the test. Coupled with rSO_2_ and angiography of collateral circulation, this patient was judged to be BTO-positive. This patient was not categorized in any Types.

## Discussion

ENPG for rNPC may involve therapeutic ICA sacrifice, which requires preoperative knowledge of the feasibility of permanent ICA occlusion (28). In comparison with abrupt ICA occlusion, the application of angiographic BTO with clinical surveillance is now a basic way to evaluate cerebral ischemic risks before permanent ICA blockage and greatly helps to reduce the incidence of postoperative cerebral infraction (13). However, BTO with clinical surveillance still has some deficiencies. For example, the clinical neurological testing is non-standardized (27) and sometimes it is difficult for clinicians to distinguish patients’ reaction following the instructions and to make verbal communication with patients due to the differences in dialects and education levels. The test of muscle strength can also be cumbersome owing to patient draping constraints. Furthermore, although BTO with clinical surveillance is sufficient in prediction of acute cerebral ischemia in awaking patients, it doesn’t work properly in evaluating delayed stroke after permanent occlusion (29). We believe that angiographic findings of collateral circulation and adjunctive blood flow measurements are quite imperative in ICA BTO.

### Useful Angiography of Collateral Circulation in Prediction of ICA BTO

It has been widely accepted that the CoW is the major and most effective collateral pathway in the brain, which can respond quickly to low perfusion areas by recruiting blood from the contralateral hemisphere *via* the anterior Circle or by recruiting blood from the posterior circulation *via* the posterior Circle to the affected hemisphere (30). In the current study, according to **Tables 1** and **2**, patients whose tested side MCA area was supplied *via* both anterior Circle and posterior Circle (Type 4) or *via* the anterior Circle alone (Type 3) demonstrated a significant reduction in BTO-positive rate and BTO false-negative rate in comparison with patients whose tested side MCA area was supplied *via* the posterior Circle alone (Type 5 + Type 6). In accordance with **Tables 1** and **2**, although we find no statistical difference of hemodynamic cerebral ischemia complication rates between Type 1 (0%) and Type 2 (67%) in Subgroup I, we speculate that the result might be caused by our limited sample size. Our findings are consistent with previous report that the anterior Circle might functionally be much more important than the posterior Circle among the primary collateral pathway (31, 32).

It is true that appearance on collateral flow can be evaluated by using the American Society of Interventional and Therapeutic Neuroradiology/Society of Interventional Radiology (ASITN/SIR) Collateral Flow Grading System for angiography (33). However, clinicians are not able to distinguish these differences clearly with their visual impression sometimes. To our experience, patients with no neurological deficits during BTO and similar hemodynamic changes like Types 5 and 6, all had collateral flow appearances of MCA areas that could be classified into ASITN/SIR Grade 3 or Grade 4, but some patients still developed delayed cerebral ischemia after therapeutic ICA occlusion.

However, reverse blood flow to tested side ICA from the posterior Circle during BTO can be easily detected in the DSA angiography. For cases among Types 5 and 6,cases without reverse blood flow to ICA suffered more from immediate or delayed cerebral ischemia by contrast with cases with reverse blood flow to ICA. So we suggest that more attention should be paid to clinical surveillance during BTO for patients with similar hemodynamic change like Types 5 and 6.We also recommend that should the sigh of reverse blood flow to tested side ICA from the posterior Circle, which indicated more than enough collateral blood perfusion, was detected among cases in Types 5 and 6, therapeutic ICA occlusion could be performed with no need to worry about delayed cerebral ischemic complications

Subgroup IV demonstrated an imbalanced anterior Circle, with one side A1 supplying more flow to an A2 than the other side A1. Relative position of A1 (dominant side) and ICA (tested side) was not associated with BTO-positive rate (*p* = 1) (**Supplemental Table 3**), while presence of the posterior Circle in Subgroup IV could reduce BTO-positive rate regardless of the relative position of A1 (dominant side) and ICA (tested side). The reason for such findings could be explained as follows: The fact that one side A1 supplying more blood flow to an A2 than the other side A1 is mainly caused by the differences in bilateral A1 diameters, with one A1 with larger diameter contributing more inflow than contralateral A1 with smaller diameter. When the collateral flow goes from the dominant side to the weak side (ICA occluded) *via* the anterior Circle, originally relatively large collateral flow will be restricted by smaller A1 according to the Pouseille equation. Reversely, when the collateral flow goes from the weak side to the dominant side (ICA occluded) *via* the anterior Circle, originally collateral blood flow remains unchanged, but the cross flow has two more ACA areas to supply compared with the other situation. One situation shows smaller blood flow to a small low perfusion area, whereas the other situation shows normal blood flow to a larger low perfusion area. Cross flow *via* the anterior Circle might not be sufficient under these two circumstances, so that’s the reason why collateral flow from the posterior Circle is indispensable for BTO-negative results in Subgroup IV. It is also crucial to point out not only BTO-positive cases but also BTO-negative cases presented themselves in Type 12. This situation could be explained by the fact that tested side MCA area could recruit enough blood perfusion *via* the anterior Circle, when there is rather little difference between A1 diameters on both sides, thus making subtle impact on cross flow *via* the anterior Circle. So, as a matter of fact, although statistical analysis shows that configurations of blood supply to tested side MCA area in BTO-negative group in Subgroup IV don’t differ between group, whose tested side ICA is contralateral to dominant A1, and group, whose tested side ICA is ipsilateral to dominant A1, we speculate that difference will occur with the rise of sample size (**Supplemental Table 2**).

Another angiographic finding highlighted was ICA stenosis contralateral to tested side. All six cases in Type 9 showed no neurological deficiencies during ICA BTO, and four cases underwent therapeutic ICA occlusion after the procedure. One (Case No. 69) of four cases presented herself with symptoms of occasional limb weakness and dizziness to our division 7 days after permanent ICA occlusion. Head CT scanning showed newly-developed lacunar infraction in the left cerebral hemisphere in contrast to previous scanning. Contralateral ICA stenosis was detected last time during BTO and we once hesitated to perform the permanent ICA occlusion, but both rSO_2_ monitoring and neurological surveillance supported the BTO-negative result. While dealing with her symptoms, we retrospectively studied the DSA angiography of the patient during BTO and considered contralateral ICA stenosis as the main reason for the newly-developed lacunar infraction. A previous study did reveal that the posterior circulation exerted a greater influence during ICA BTO in patients with high-grade contralateral ICA stenosis (31). Combining previous report with our case, we presume that ipsilateral posterior Circle plays a much more quantitatively significant role in protecting the hemisphere against hemodynamic ischemia. Contralateral ICA stenosis might still enable patients to narrowly tolerate temporary ICA sacrifice in resting state, but it does have an impact on the capacity to supply blood to the affected vascular area *via* the anterior Circle at times of stress.

For another, besides collateral flow through CoW, two of six rNPC patients with CICAO on the lesion side didn’t have any cerebral ischemic symptoms and have gradually developed secondary collateral pathway, such as ophthalmic arteries and leptomengningeal arteries, to the low perfusion area (**Supplemental Table 4**). However, Case No. 58 in the Case Illustration, who developed collateral flow *via* leptomengningeal branches from PCA to the tested side MCA area without the presence of PcomA, was diagnosed with acute cerebral hemodynamic ischemia owing to drowsiness, decrease in muscle strength and simultaneous significant reduction in tested side rSO_2_ during the procedure. Furthermore, Case No. 62 in Type 7 not only gained its collateral flow *via* the posterior Circle on the tested side, but also *via* leptomengningeal arteries contralateral to the tested side. The rSO_2_ monitoring of this case also showed no abnormal fluctuation on tested side. Evidence above demonstrates the fact that secondary collateral pathways, which include but are not limited to extracranial-intracranial anastomoses *via* the meningeal or ophthalmic arteries and pial collaterals, need more time to function (34, 35). Based on our study, secondary collateral pathways usually present themselves as a substitution when the primary collateral pathways fall short or a supplement when the primary collateral pathways are insufficient. The presence of secondary collateral pathways alone is often linked with BTO-positive results, because it is considered to be a sign of deteriorated hemodynamic condition of the brain in acute ischemic phase.

Case No. 63 in Type 8 was found cfPCA and A1 absence ipsilateral to the tested side during ICA BTO and was regarded as BTO-positive 10 min after the balloon was inflated. Since the patient didn’t have a complete anterior and posterior Circle, as a matter of fact, we could have predicted the intolerance of ICA sacrifice of this case earlier, should we had performed head CTA/MRA or Matas maneuver (angiography of the non-tested ICA during manual carotid compression on the tested side) and Allcock maneuver (angiography of the vertebral artery during manual carotid compression on the tested side) before ICA BTO. What also needed to mention was that another seven BTO-positive cases(Case Nos. 81–87) in our study were excluded from all the Subgroups,as they failed to perform angiography of collateral flow in time. For patients those who quickly fail the BTO, earlier awareness of their CoW development might spare them from unnecessary BTO. Anyway, though BTO is a simple procedure, it was reported to increase the rate of complications for neuroangiography from 1.3% (36) to 3 to 4% (16, 37). Unfortunately, we didn’t perform head CTA/MRA or Matas maneuver and Allcock maneuver before the procedure in this study. However, these exams and operations might help to reduce overall cost and complication rate for each individual.

### Suitable Adjunctive Techniques Chosen for Selected Cases During ICA BTO

When it came to the adjunctive techniques during BTO, we chose NIRS cerebral oximeter to monitor rSO_2_ change during BTO. rSO_2_ monitoring is a quantitative method that also carries the merits of being noninvasive and cost-effective. The baseline values of rSO_2_ in our study ranged from 64 to 80% (mean 72.3 ± 5.1%) and was similar to previous reports (18, 38). Many scholars have applied rSO_2_ monitoring during temporary ICA occlusion for the detection of cerebral ischemia. However, a critical ΔrSO_2_ level to induce neurological deficit has not been well established yet. Our study revealed that the most suitable cut-off value detecting the BTO-positive group was 5%. All the cases regarded as BTO-positive by clinical surveillance showed an irreversible drop by at least 5% in rSO_2_ on the tested side. However, comparison of change in rSO_2_ between BTO false-negative group and BTO negative group showed no statistical difference. Furthermore, all BTO false-positive cases didn’t showed obvious rSO_2_ fluctuation during BTO in our study. These evidences supported our experience that rSO_2_ monitoring didn’t work well in detecting delayed cerebral ischemia after BTO. The fact that the sensors can only be put on the forehead of both sides and monitor limited to the frontal lobe may greatly affect the sensitivity and specificity in sorting out BTO false-negative cases.

That is why we consider that other more quantitative blood flow measurements, such as CTP, Technetium-99m hexamethylpropyleneamine oxime (^99m^Tc-HMPAO), single-photon emission CT(SPECT) and xenon-enhanced CT(XeCT), might be essential for cases with certain kind of collateral flow configurations during BTO. According to our study, clinically BTO-negative cases, who recruit collateral flow *via* both primary and secondary pathways (e.g. Case No. 62), or who have similar hemodynamic changes like Types 5 and 6 (e.g. Case No. 50), or who have contralateral ICA stenosis (e.g. Case No. 69) are proper candidates for BTO in conjunction with these more quantitative blood flow measurements before permanent ICA occlusion. In fact, cases, whose angiography of collateral circulation doesn’t fully support clinically BTO-negative results, are recommended to use more quantitative blood flow measurements to avoid delayed cerebral ischemia after permanent ICA occlusion. Tomoyoshi Kuribara et al. reported that patients whose MTT obtained through CT perfusion at less than 16.4% increase compared with that on the contralateral side might be treated with abrupt ICA occlusion (38). Quantitative CBF studies using XeCT pointed out that a flow of less than 30 ml/100 g/mg as the threshold for occurrence of neurological symptoms after ICA sacrifice (13, 39). However, BTO false-negative rate has also been reported in these method,with ^99m^Tc-HMPAO studies up to 20% (13, 40, 41) and XeCT up to 3–10% (13, 39, 42). It has also been suggested that SPECT asymmetry analysis carries a high rate of BTO false-positive test results (43, 44). Despite the fact that room to room transfer, extended test time and complex operation are needed for these equipment, we believe more detailed information of cerebral hemodynamic changes during ICA BTO will decrease BTO false-negative rate to some extent.

Method chosen for distinguishing BTO results greatly influence the following treatment of patients with rNPC. For patients whom clinicians falsely think that will develop delayed cerebral stroke, patients will undergo covered stent implantation of ICA before ENPG. This might increase perioperative complication rate of a hurried ENPG caused by routine antiplatelet treatment after covered stent implantation, as rNPC patients want the surgery urgently and have little time to waste. That was also the reason why we didn’t choose hypotensive challenge or neurophysiologic monitoring as adjunctive techniques, as such tests would possibly have withheld ICA sacrifice that actually was feasible (28, 45).

Overall, all adjunctive techniques coupled with ICA BTO have their own pearls and pitfalls, the results of ICA BTO need a comprehensive judgement.

### Study Limitations

This study had several limitations. First, it was a retrospective study with limited samples in each subgroups and types. We didn’t observe the long nature history of cases with therapeutic ICA occlusion, as patients were discharged from our neurosurgical division soon after the treatment and received ENPG. Secondly, all the BTO false-negative cases in our study only presented with symptomatic ischemic events. The following head CT scanning confirmed the diagnose of delayed stroke. However, there is cerebral ischemia identified radiographically that is asymptomatic, so it might be essential to perform diffusion-weighted or FLAIR MRI imaging before BTO and after therapeutic ICA occlusion. Thirdly, collateral angiography could not be performed in some patients owing to ischemic symptoms during temporal occlusion of the ICA. The data of these extremely poor collateral configurations were missing. In addition, some patients needed BTO alone for their low risk of ICA injury according to a new assessment of ICA invasion (46). This might also influence the detection of BTO false-negative cases and results. As a consequence of these limitations, it’s necessary to confirm our results with prospective and large sample studies.

## Conclusion

CTA/MRA scanning of the brain with Matas and Allcock maneuvers before ICA BTO is essential. Angiographic findings before ICA BTO and angiography of cerebral collateral circulation during ICA BTO are significantly correlated with ICA BTO results. Angiographic ICA BTO can be performed in conjunction with NIRS cerebral oximeter for its advantage of being noninvasive, real-time, cost-effective, simple for operation and most importantly for its correct prediction of rSO_2_ outcome of ICA sacrifice. However, in order to ensure a safe carotid artery occlusion, more quantitative adjunctive blood flow measurements are recommended when angiography of cerebral collateral circulation doesn’t fully support rSO_2_ outcome among clinically ICA BTO-negative cases.

## Data Availability Statement

The original contributions presented in the study are included in the article/**Supplementary Material**. Further inquiries can be directed to the corresponding authors.

## Ethics Statement

The studies involving human participants were reviewed and approved by the ethics committees of Renji Hospital, School of Medicine, Shanghai Jiao Tong University. This was a retrospective study, so informed patient consent was not required.

## Author Contributions

JD, YQ, RY, and BC conceived, designed and supervised the study. RY, HW, and BC wrote the manuscript. RY, WS, XH, TW, and YG collected and analyzed the data. RY and BC revised the manuscript All authors contributed to the article and approved the submitted version.

## Funding

This work was supported by Shanghai 2020 ‘Science and Technology Innovation Action Plan’ Medical Innovation Research Special Project (20Y11905900), National Natural Science Foundation of China (81874215), and Natural Science Research Project of Minhang District (Shanghai) (2019MHZ090).

## Conflict of Interest

The authors declare that the research was conducted in the absence of any commercial or financial relationships that could be construed as a potential conflict of interest.

The reviewer BL declared a shared affiliation with the authors to the handling editor at time of review.
