# Supplementary material for: Balloon Test Occlusion of Internal Carotid Artery in Recurrent Nasopharyngeal Carcinoma Before Endoscopic Nasopharyngectomy: A Single Center Experience
Source: Front Oncol. 2021 Jul 6;11:674889. doi: 10.3389/fonc.2021.674889 (PMC8290142; doi:10.3389/fonc.2021.674889)
Supplement: Supplementary file 1 [file DataSheet_1.docx]

Supplementary Material

**Supplemental Table 1** Clinical features of 87 cases with BTO

| Case  no. | Age(y),  sex | △rSO_2_(%)，  initial rSO_2_(%) | Tested side  ICA(Left/Right) | Treatment of  ICA | Source of collateral flow to MCA  (CoW,ipsilateral pial artery,etc ) | BTO  result | Cerebral ischemic  complications |
| --- | --- | --- | --- | --- | --- | --- | --- |
| SubgroupI |  |  |  |  |  |  |  |
| Type1 |  |  |  |  |  |  |  |
| 1 | 51,M | -4,77 | R | Occlusion | AC | － | N |
| 2 | 49,M | -2,81 | R | BTO alone | AC | － | N |
| 3 | 58,M | -1,72 | L | Occlusion | AC | － | N |
| 4 | 58,M | -2,71 | R | BTO alone | AC | － | N |
| Type2 |  |  |  |  |  |  |  |
| 5 | 68,M | –––– | L | BTO alone | PC | ＋ | Right side muscle force decreased, Lethargy |
| 6 | 40,M | -2,79 | L | BTO alone | PC | － | N |
| 7 | 58,F | -5,78 | L | BTO alone | PC | ＋ | Right side muscle force decreased,  Lethargy |
| SubgroupII |  |  |  |  |  |  |  |
| Type3 |  |  |  |  |  |  |  |
| 8 | 45,M | -1,75 | R | Occlusion | AC | － | N |
| 9 | 49,M | -2,78 | L | Occlusion | AC | － | N |
| 10 | 50,M | –––– | L | Occlusion | AC | － | N |
| 11 | 54,F | –––– | L | Occlusion | AC | － | N |
| 12 | 68,M | -1,73 | L | Occlusion | AC | － | N |
| 13 | 63,M | –––– | R | Occlusion | AC | － | N |
| 14 | 57,M | 0，72 | R | Occlusion | AC | － | N |
| 15 | 52,M | –––– | L | Occlusion | AC | － | N |
| 16 | 50,M | –––– | R | Occlusion | AC | － | N |
| 17 | 47,M | -2,86 | L | Occlusion | AC | － | N |
| 18 | 51,M | -3,77 | L | BTO alone | AC | － | N |
| 19 | 30,F | 0,76 | R | BTO alone | AC | － | N |
| 20 | 60,F | –––– | R | BTO alone | AC | － | N |
| 21 | 37,M | –––– | R | BTO alone | AC | － | N |
| 22 | 57,M | -1,69 | R | BTO alone | AC | － | N |
| 23 | 63,F | -1,78 | L | BTO alone | AC | － | N |
| Type4 |  |  |  |  |  |  |  |
| 24 | 49,F | -3,72 | R | Occlusion | AC+PC | － | N |
| 25 | 58,F | -5,77 | L | Occlusion | AC+PC | － | N |
| 26 | 42,M | –––– | L | Occlusion | AC+PC | － | N |
| 27 | 52,M | -4,70 | L | Occlusion | AC+PC | － | N |
| 28 | 60,M | –––– | L | Occlusion | AC+PC | － | N |
| 29 | 56,M | -3,71 | R | Occlusion | AC+PC | － | N |
| 30 | 54,M | –––– | L | Occlusion | AC+PC | － | N |
| 31 | 39,M | -1,77 | R | Occlusion | AC+PC | － | N |
| 32 | 42,M | –––– | L | Occlusion | AC+PC | － | N |
| 33 | 45,M | -3,77 | R | Occlusion | AC+PC | － | N |
| 34 | 44,M | -2,76 | L | Occlusion | AC+PC | － | N |
| 35 | 48,M | -2,76 | L | Occlusion | AC+PC | － | N |
| 36 | 46,M | -3,76 | R | BTO alone | AC+PC | － | N |
| 37 | 48,M | –––– | L | BTO alone | AC+PC | － | N |
| 38 | 48,M | –––– | L | BTO alone | AC+PC | － | N |
| 39 | 37,M | 0,75 | L | BTO alone | AC+PC | － | N |
| 40 | 57,M | 0,68 | L | BTO alone | AC+PC | － | N |
| 41 | 58,M | -5,75 | R | BTO alone | AC+PC | － | N |
| 42 | 58,M | -2,74 | L | BTO alone | AC+PC | － | N |
| 43 | 37,M | -2,74 | R | BTO alone | AC+PC | － | N |
| 44 | 37,M | 0,73 | L | BTO alone | AC+PC | － | N |
| 45 | 53,M | -6,72 | L | BTO alone | AC+PC | － | N |
| 46 | 49,F | -3,75 | R | BTO alone | AC+PC | － | N |
| 47 | 49,F | -1,77 | L | BTO alone | AC+PC | － | N |
| 48 | 53,M | –––– | R | BTO alone | AC+PC | － | N |
| 49 | 45,M | –––– | L | BTO alone | AC+PC | － | N |
| Type5 |  |  |  |  |  |  |  |
| 50 | 42,M | -2,74 | L | Occlusion | PC | －＋ | Aphasia,  Hemiplegia of right limb |
| 51 | 38.M | -3,70 | L | Occlusion | PC | － | N |
| 52 | 40,M | -1,80 | L | Occlusion | PC | － | N |
| 53 | 55,M | –––– | L | Occlusion | PC | － | N |
| 54 | 54,F | -9,80 | R | BTO alone | PC | ＋ | Left side muscle force decreased,  Lethargy |
| 55 | 61,M | -7,64 | R | BTO alone | PC | ＋ | Left side muscle force decreased,  Lethargy |
| 56 | 51,M | -6,78 | R | BTO alone | PC | － | N |
| 57 | 53,M | –––– | R | BTO alone | PC | － | N |
| Others |  |  |  |  |  |  |  |
| 58 | 61,M | -7,71 | R | BTO alone | PCA | ＋ | Slow response |
| SubgroupIII |  |  |  |  |  |  |  |
| Type6 |  |  |  |  |  |  |  |
| 59 | 62,F | -5,77 | L | BTO alone | PC | － | N |
| 60 | 51,F | 0,78 | R | BTO alone | PC | － | N |
| 61 | 64,M | -3,77 | R | Occlusion | PC | －＋ | Left side muscle force decreased,  Dizziness |
| Type7 |  |  |  |  |  |  |  |
| 62 | 42,F | -1,70 | L | BTO alone | PC | － | N |
| Type8 |  |  |  |  |  |  |  |
| 63 | 59,M | -5,77 | R | BTO alone | ACA | ＋ | Lethargy |
| SubgroupIV |  |  |  |  |  |  |  |
| Type9 |  |  |  |  |  |  |  |
| 64 | 58,F | -2,88 | R | Occlusion | AC+PC | － | N |
| 65 | 48,M | -4,74 | L | BTO alone | AC+PC | － | N |
| 66 | 47,M | 0,67 | L | BTO alone | AC+PC | － | N |
| 67 | 46,M | –––– | L | Occlusion | AC+PC | － | N |
| 68 | 60,F | -5,78 | L | BTO alone | AC+PC | － | N |
| 69 | 68,F | -4,82 | L | Occlusion | AC+PC | －＋ | Right side muscle force decreased,  Dizziness |
| Type10 |  |  |  |  |  |  |  |
| 70 | 41,F | –––– | R | Occlusion | AC+PC | － | N |
| 71 | 28,F | 0,63 | R | Occlusion | AC+PC | － | N |
| 72 | 51,F | -5,74 | L | Occlusion | AC+PC | － | N |
| 73 | 57,M | –––– | R | BTO alone | AC+PC | － | N |
| 74 | 70,F | -2,59 | R | Occlusion | AC+PC | － | N |
| Type11 |  |  |  |  |  |  |  |
| 75 | 65,M | -6,81 | L | BTO alone | AC+PCA | ＋ | Slight aphasia,  Lethargy |
| Type12 |  |  |  |  |  |  |  |
| 76 | 63,M | -8,71 | R | BTO alone | AC | ＋ | Slow response,  Right side muscle force decreased |
| 77 | 55,M | 4,77 | L | BTO alone | AC | ＋ | Headache,  Right side muscle force decreased |
| 78 | 62,M | -3,70 | L | Occlusion | AC | － | N |
| 79 | 69,M | 0,71 | R | Occlusion | AC | － | N |
| Others |  |  |  |  |  |  |  |
| 80 | 63,M | -10,69 | L | Covered stent | –––– | ＋ | Loss of consciousness |
| Cases without cerebral collateral angiography |  |  |  |  |  |  |  |
| 81 | 67,M | –––– | L | BTO alone | –––– | ＋ | Loss of consciousness |
| 82 | 62,F | -9,77 | L | BTO alone | –––– | ＋ | Loss of consciousness |
| 83 | 69,M | –––– | L | BTO alone | –––– | ＋ | Loss of consciousness |
| 84 | 33,F | –––– | R | BTO alone | –––– | ＋ | Loss of consciousness |
| 85 | 61,M | -7,73 | R | BTO alone | –––– | ＋ | Loss of consciousness |
| 86 | 68,M | -7,72 | R | BTO alone | –––– | ＋ | Loss of consciousness |
| 87 | 51,M | –––– | R | BTO alone | –––– | ＋ | Loss of consciousness |

AC:the anterior Circle; PC:the posterior Circle; PCA:posterior cerebral artery; ACA:anterior cerebral artery; N:No cerebral ischemic complications;

––––:no rSO_2_ monitoring or no angiography of collateral circulation; ＋:BTO-positive; －:BTO-negative; －＋:BTO false-negative;

**Supplemental Table 2** The summary of the relationship between relative position of A1(dominant side) and ICA (tested side) and its tested side MCA area blood supply in BTO-negative groups in Subgroup IV

|  | Relative position of A1(dominant side) and ICA (tested side) | | |
| --- | --- | --- | --- |
| Tested side MCA area blood supply | Contralateral (%)  (n=7) | Ipsilateral (%)  (n=5) | Total (%)  (n=12) |
| AC | 2 (17) | 0 (0) | 2(17) |
| AC+PC | 5 (42) | 5(42) | 10(83) |
| Total | 7 (58) | 5 (42) | 12 (100) |

AC: the anterior Circle；PC: the posterior Circle

**Supplemental Table 3** The summary of the relationship between relative position of A1(dominant side) and ICA (tested side) and its BTO results (Subgroup IV)

| Relative position of A1(dominant side) and ICA (tested side) | BTO-positive (%)  (n=4) | BTO-negative (%)  (n=12) | Total (%)  (n=16) |
| --- | --- | --- | --- |
| Contralateral | 2 (13) | 7 (44) | 9 (56) |
| Ipsilateral | 2 (13) | 5 (31) | 7 (44) |
| Total | 4 (25) | 12 (75) | 16 (100) |

**Supplemental Table 4** Clinical features of 6 patients with chronic internal carotid artery occlusion (CICAO)

| Patient  No. | Age(year),  Gender | Lesion  Side | Source of collateral ﬂow | |
| --- | --- | --- | --- | --- |
|  |  |  | ipsilateral ACA area | ipsilateral MCA area |
| 1 | 47,M | Left | AC | AC |
| 2 | 41,F | Right | AC | AC+PC |
| 3 | 31,M | Right | AC | AC |
| 4 | 39,M | Left | AC | PC+OphA |
| 5 | 51,M | Right | AC | AC |
| 6 | 71,F | Left | AC | ACA+PCA |

AC: the anterior Circle；PC: the posterior Circle; ACA:anterior cerebral artery; PCA:posterior cerebral artery; OphA:Ophthalmic Artery
